# Genome-Wide Identification and Expression Profiling of Sugar Transporter Protein (STP) Family Genes in Cabbage (*Brassica oleracea* var. *capitata* L.) Reveals their Involvement in Clubroot Disease Responses

**DOI:** 10.3390/genes10010071

**Published:** 2019-01-21

**Authors:** Wei Zhang, Shenyun Wang, Fangwei Yu, Jun Tang, Li Yu, Hong Wang, Jianbin Li

**Affiliations:** Jiangsu Key Laboratory for Horticultural Crop Genetic Improvement, Institute of Vegetable Crops, Jiangsu Academy of Agricultural Sciences, Nanjing 210014, China; zhangwei@jaas.ac.cn (W.Z.); wangshenyun@jaas.ac.cn (S.W.); yfw@jaas.ac.cn (F.Y.); tj@jaas.ac.cn (J.T.); scyuli@jaas.ac.cn (L.Y.); wanghong@jaas.ac.cn (H.W.)

**Keywords:** Cabbage (*Brassica oleracea* var. *capitata* L.), expression profile, phylogenetic analysis, clubroot disease response, sugar transporter protein (STP)

## Abstract

Sugar transporter protein (*STP*) genes are involved in multiple biological processes, such as plant responses to various stresses. However, systematic analysis and functional information of *STP* family genes in *Brassica oleracea* are very limited. A comprehensive analysis was carried out to identify *BoSTP* genes and dissect their phylogenetic relationships and to investigate the expression profiles in different organs and in response to the clubroot disease. A total of 22 *BoSTP* genes were identified in the *B. oleracea* genome and they were further classified into four clades based on the phylogenetic analysis. All the BoSTP proteins harbored the conserved sugar transporter (Sugar_tr, PF00083) domain, and the majority of them contained 12 transmembrane helices (TMHs). Rates of synonymous substitution in *B. oleracea* relative to *Arabidopsis thaliana* indicated that *STP* genes of *B. oleracea* diverged from those of *A. thaliana* approximately 16.3 million years ago. Expression profiles of the *BoSTP* genes in different organs derived from RNA-Seq data indicated that a large number of the *BoSTP* genes were expressed in specific organs. Additionally, the expression of *BoSTP4b* and *BoSTP12* genes were induced in roots of the clubroot-susceptible cabbage (CS-JF1) at 28 days after inoculation with *Plasmodiophora brassicae*, compared with mock-inoculated plants. We speculated that the two BoSTPs might be involved in monosaccharide unloading and carbon partitioning associated with *P. brassicae* colonization in CS-JF1. Subcellular localization analysis indicated that the two BoSTP proteins were localized in the cell membrane. This study provides insights into the evolution and potential functions of *BoSTPs*.

## 1. Introduction

Sugars (e.g., monosaccharides, sucrose, and polyols) act as carbohydrate molecules, main energy sources, precursors of cellular compounds, and signaling molecules for signal transduction as well as environmental stress responses, which are important for plant growth and development [1,2,3,4]. Sugars are mainly synthesized in leaves (source organs) and then translocated via phloem sap into the sink organs, such as modified leaves, roots, seeds, fruits, and other reproductive organs [5,6]. In plants, sugar transport is mediated by monosaccharide transporters (MSTs) and sucrose transporters (SUTs) and sugars will eventually be exported transporters (SWEETs) [7,8]. The sucrose can be transported from the phloem to sink cells via a symplastic pathway or an apoplastic pathway [6]. Apart from sucrose, the transport of glucose and fructose, which are hydrolyzed from the sucrose in the apoplast, is regulated by sugar transporter proteins (STPs) and hexose transporters (HTs) [9,10].

Sugar transporter proteins, belonging to the MST superfamily, commonly contain 12 transmembrane helices (TMHs) and are localized in the cell membrane [11]. Sugar transporter proteins are also regarded as H^+^/sugar symporters and can transport fructose, glucose, galactose, mannose, and xylose [12]. With the rapid development of whole-genome sequences, genome-wide identification of *STP* genes in various plant species have been reported, such as *Arabidopsis thaliana* [12], cassava (*Manihot esculenta*) [13], grape (*Vitis vinifera*) [14], rice (*Oryza sativa*) [15], tomato (*Solanum lycopersicum*) [16], pear (*Pyrus bretschneideri* Rehd) [17], and woodland strawberry (*Fragaria vesca*) [18]. In *Arabidopsis*, a total of 14 STP proteins (AtSTP1-14) have been identified and they were found to mediate the uptake of hexoses from the apoplastic space across the cell membrane [19,20]. In addition, AtSTP1 is the first monosaccharide transporter identified and it is expressed in guard cells of cotyledons, rosette leaves, sepals, ovaries, and stems [21,22]. Heterologous expression indicated that AtSTP1 protein is a high-affinity monosaccharide/H^+^ symporter and is able to transport a suite of hexoses, but not fructose [23]. Moreover, AtSTP4 protein is also a high-affinity hexose transporter and the *AtSTP4* gene is expressed in leaves, root tips, and pollen tubes. The expression level of *AtSTP4* is strongly increased in response to pathogen attack and wounding [24]. Furthermore, in response to powdery mildew infection, the *AtSTP4* and the invertase gene, *Atβfruct1*, are coordinately expressed [25]. In addition, among the 14 characterized AtSTP proteins, all of them are high-affinity hexose transporters except AtSTP3, which is a low-affinity hexose transporter and is expressed in green leaves, but not in a sink organ [19]. Interestingly, the expression of the *AtSTP3* gene is also induced by pathogen attack and wounding [20]. Although the expression profiles and functional analysis of *AtSTP* genes in *Arabidopsis* have been explored, the extensive expression profiles of the *BoSTP* genes in cabbage remain poorly characterized.

Cabbage (*Brassica oleracea* var. *capitata* L.) is one of the most economically important leafy vegetables worldwide. The harvested area of cabbages and other *Brassicas* was 2,513,707 ha in 2017, with an annual yield of 71.45 million tons [26]. Clubroot disease is a soil-borne disease caused by the obligate biotrophic protest, *Plasmodiophora brassicae*. *Plasmodiophora brassicae* can infect almost all Brassicaceae crops, and is one of the most devastating plant diseases in the world [27,28]. Clubroot disease is known to occur in more than 60 countries and results in a 10–15% reduction in yields on a global scale [27]. It is estimated that 3.2–4.0 million ha of Brassicaceae crops are infected by clubroot pathogen annually, accounting for more than one third of the total cultivation regions of Brassicaceae crops [29]. The life cycle of *P. brassicae* consists of three distinct stages: The survival of resting spores in the soil, the primary infection (root hair infection), and the secondary infection (root cortex infection) [30,31,32]. The survival resting spores germinate to release the primary zoospores and penetrate the root hairs to form primary plasmodia [33]. The primary plasmodia undergo a series of cell divisions to form secondary zoospores [34]. Then, the secondary zoospores form multinucleate secondary plasmodia within the root cortex, which leads to cell hypertrophy and hyperplasia in the cortex and stele, resulting in the development and formation of galls. Finally, after the galls disintegrate, the resting spores cleaved from the secondary plasmodia are released into the soil to complete the disease cycle [35]. The resting spores can survive in the soil for 6–12 years, making this clubroot disease hard to control once the soil is contaminated [36].

In this study, we performed a genome-wide analysis of the *STP* family genes in *B. oleracea*, and shed light on their gene structure, chromosomal localization, transmembrane regions, and conserved motifs. The expression profiles of *BoSTP* genes in different organs and in response to clubroot disease were analyzed using the RNA-Seq data, in an attempt to understand their possible roles in clubroot disease resistance.

## 2. Materials and Methods

### 2.1. Identification of Sugar Transporter Proteins in Brassica oleracea

The *B. oleracea* genome sequence, downloaded from the *B. oleracea* genome database (http://ocri-genomics.org/bolbase/), was used to identify the *BoSTP* genes [37]. The genome sequence of *Brassica rapa* was obtained from the *Brassica* database (http://brassicadb.org/brad/) [38]. The *AtSTP* gene sequences, downloaded from The Arabidopsis Information Resource (TAIR) database (http://www.arabidopsis.org/), were used as the seed sequences to search the orthologous and syntenic paralogous *STP* genes in *B. oleracea* and *B. rapa* using the online tool (http://brassicadb.org/brad/searchSyntenytPCK.php). The hmmscan tool [39] with the ‘gathering threshold’ and the SMART tool [40] were then used to predict the functional domains of the potential STP proteins. The identified STPs without the Sugar_tr domain (PF00083) were filtered out. The naming of STP proteins from *B. oleracea* and *B. rapa* was based on sequence similarity to the corresponding AtSTPs. 

### 2.2. Characterization and Phylogenetic Analysis of BoSTPs

The ProtParam tool (https://web.expasy.org/protparam/) was used to analyze the physical and chemical parameters of the BoSTP proteins, including the molecular weight, theoretical isoelectric point (pI), instability index, aliphatic index, and grand average of hydropathicity (GRAVY). The Gene Structure Display Server 2.0 (GSDS 2.0, http://gsds.cbi.pku.edu.cn/) was used to generate a schematic diagram of the exon and intron organization by comparing the genome sequence and the corresponding coding sequence (CDS) of each *BoSTP* gene [41]. Multiple alignment of BoSTP, BrSTP, and AtSTP protein sequences was performed using ClustalW [42], and the maximum likelihood (ML) phylogenetic tree was constructed using MEGA7 with the bootstrap of 1000 replicates [43].

### 2.3. Chromosomal Localization and the Calculation of Ka/Ks

The MapChart 2.30 software [44] was used to determine the chromosomal localization of the *BoSTP* genes. The online tool (http://brassicadb.org/brad/searchSyntenytPCK.php) was used to identify orthologs and paralogs of the *STP* genes in *B. oleracea* and *B. rapa* [45]. The relationships of orthologs and paralogs were plotted using the Circos software [46]. To check whether positive selection has driven the evolution of *STP* genes, the synonymous substitution rate (*Ks*) and nonsynonymous substitution rate (*Ka*) values of orthologous genes were calculated using the DnaSP 6 software [47]. A *Ka*/*Ks* ratio greater than 1 indicates a positive selection of the gene pairs, less than 1 indicates a purifying selection, while approximately equal to 1 indicates a neutral evolution. The *Ks* values of syntenic orthologs between *BoSTPs* and *AtSTPs* were plotted as the density using an R package [48]. The divergence time was computed as T = *Ks*/(2 × 1.5 × 10^−8^) × 10^−6^ million years ago (MYA) [49].

### 2.4. Prediction of TMH, Putative Functional Domain, and Subcellular Localization

The TMHs of BoSTP proteins were predicted by the TMHMM Server v. 2.0 (http://www.cbs.dtu.dk/services/TMHMM/). The putative functional domains of the identified BoSTP proteins were predicted by hmmscan [39] and SMART [40]. The subcellular localization of STP proteins was predicted using Plant-mPLoc [50].

### 2.5. Expression Profile Analysis of BoSTP Genes

To analyze the expression profiles of *BoSTP* genes in different organs and in response to *P. brassicae* infection, RNA-Seq data of GSE42891 (GEO database) submitted by Liu et al. [37] and PRJNA453960 (BioProject accession) submitted by Chinese Academy of Agricultural Sciences (CAAS) were downloaded from the National Center for Biotechnology Information (NCBI, https://www.ncbi.nlm.nih.gov/). The GSE42891 dataset included gene expression profiles of seven different organs (bud, callus, flower, leaf, root, silique, and stem) in cabbage homozygous line 02-12 [37]. The PRJNA453960 dataset contained gene expression profiles of roots in clubroot-resistant cabbage Xiangan336 (CR-XG336) and clubroot-susceptible cabbage Jingfeng No. 1 (CS-JF1) after the inoculation with *P. brassicae* spores suspended in water at 7 (primary infection stage) and 28 (clubroot formation stage) days after inoculation (DAI), respectively. Furthermore, the corresponding root samples at 7 DAI and 28 DAI without inoculation were sampled as mock controls. Raw RNA-Seq reads with three biological replicates from the PRJNA453960 dataset were processed to trim the adapter and low-quality sequences using Trimmomatic [51]. The high-quality reads were aligned to the *B. oleracea* genome database using HISAT [52] allowing up to 3 edit distances. Following the alignments, raw counts for each gene were derived and normalized into FPKM (fragments per kilobase of exon model per million mapped fragments). Raw count data was then fed to DESeq2 [53] to identify differentially expressed genes with a cutoff of fold change >2 and FDR <0.05. The heatmap of *BoSTP*s expression profiles was generated using the R package pheatmap (http://rpackages.ianhowson.com/cran/pheatmap/) based on the log2 transformed FPKM values.

### 2.6. Subcellular Localization Analysis of BoSTPs in Tobacco

To investigate the subcellular localization of BoSTPs, transient transformation of translational GFP fusion vectors into tobacco leaf epidermal cells was used. The full-length CDS of *BoSTP4b* and *BoSTP12* genes were amplified with the forward primer containing a *Sma* I restriction site and the reverse primer containing a *Xba* I restriction site. The primer sequences are listed in Supplementary File S1. The reaction mixture (50 µL) of the gene amplification consisted of 1 × PCR buffer (final concentrations, 10 mM Tris-HCl, 2.5 mM MgCl2, and 50 mM KCl [pH 8.3]), 0.2 mM dNTP, 0.5 µM forward and reverse primer, 50 ng of cDNA template and 2.5 U of Taq DNA polymerase (TaKaRa, Dalian, China). The amplification was performed at 95 °C for 3 min, followed by 35 cycles of 94 °C for 30 s, 56 °C for 30 s, and 72 °C for 50 s, with a final extension at 72 °C for 7 min and hold at 4 °C. The amplification products were digested with *Sma* I and *Xba* I, and ligated into the pCAMBIA1300-35S-GFP (35S-GFP) vector for constructing the translational GFP fusion constructs. The recombined plasmids were then transformed into *Agrobacterium tumefaciens* strain GV3101 [54]. The methods of preparing *A. tumefaciens* for transient expression and infiltrating into tobacco epidermal cells were carried out according to the previous protocols [55]. Leaves transformed with the vector of 35S-GFP (GFP alone) was used as the control. The fluorescence and bright-light images of transiently infected tobacco leaves were observed under a fluorescence microscope (BX41, Olympus, Rungis, France) after 48 h of infiltration.

## 3. Results

### 3.1. Identification and Phylogenetic Analysis of STPs in Brassica oleracea, Brassica rapa, and Arabidopsis thaliana

**Figure 1:**
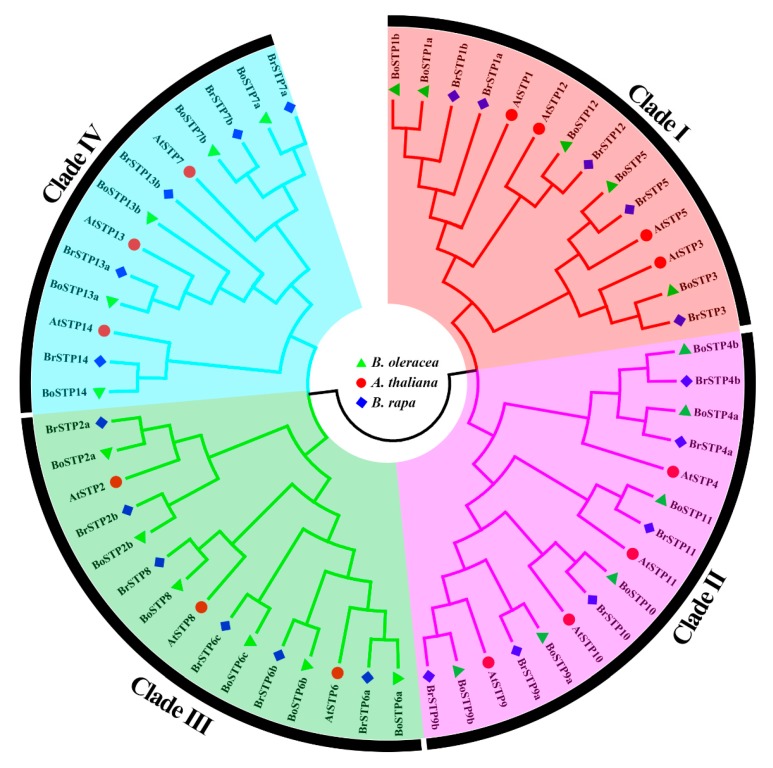
Phylogenetic tree of *B. oleracea*, *Brassica rapa*, and *A. thaliana* STP proteins. Phylogenetic analysis of 58 STP proteins from *B. oleracea* (22), *B. rapa* (22), and *A. thaliana* (14) showing similar groups in the three species. Four clades were marked with different background colors.

In the present study, a total of 22 BoSTP proteins and 22 BrSTP proteins were identified in the *B. oleracea* and *B. rapa* genomes, respectively (Supplementary File S2). These proteins were named BoSTP1 to BoSTP14 and BrSTP1 to BrSTP14, respectively, according to their *A. thaliana* homologs (Table 1). An unrooted phylogenetic tree of 58 STP proteins from *B. oleracea*, *B. rapa*, and *A. thaliana* was constructed to elucidate their evolutionary relationships (Figure 1). Phylogenetic analysis revealed that these STP proteins could be divided into four distinct clades (I–IV), and the BoSTPs were grouped with AtSTPs and BoSTPs in each clade. The clade I, II, III, and IV contained 14, 16, 15, and 13 members, respectively. Physical and chemical characteristics of BoSTP proteins were predicted. The length of the BoSTP proteins ranged from 345 amino acids (AA, BoSTP14) to 532 AA (BoSTP13b), with the corresponding open reading frames (ORFs) ranging from 1038 bp to 1599 bp (Table 1). The molecular weights ranged from 38.33 kDa (BoSTP14) to 58.04 kDa (BoSTP13b), and the theoretical isoelectric points (pI) ranged from 5.79 (BoSTP11) to 9.31 (BoSTP13a) (Table 1). In addition, the instability index, aliphatic index and GRAVY of BoSTP proteins ranged from 30.06 to 45.45, 97.50 to 112.94, and 0.45 to 0.69, respectively (Table 1). 

### 3.2. Chromosomal Distribution of BoSTP Genes

**Figure 2:**
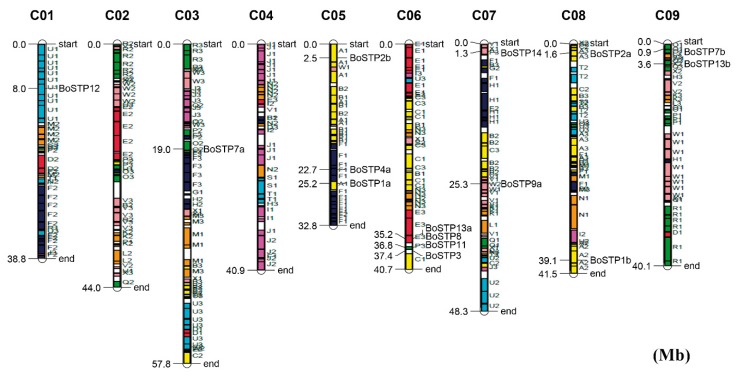
Distribution of *BoSTP* genes on the nine chromosomes of *B. oleracea*. The 24 conserved collinear blocks on each chromosome are labeled A–X and the three sub-genomes are plotted, based on a previous report [37].

**Figure 3:**
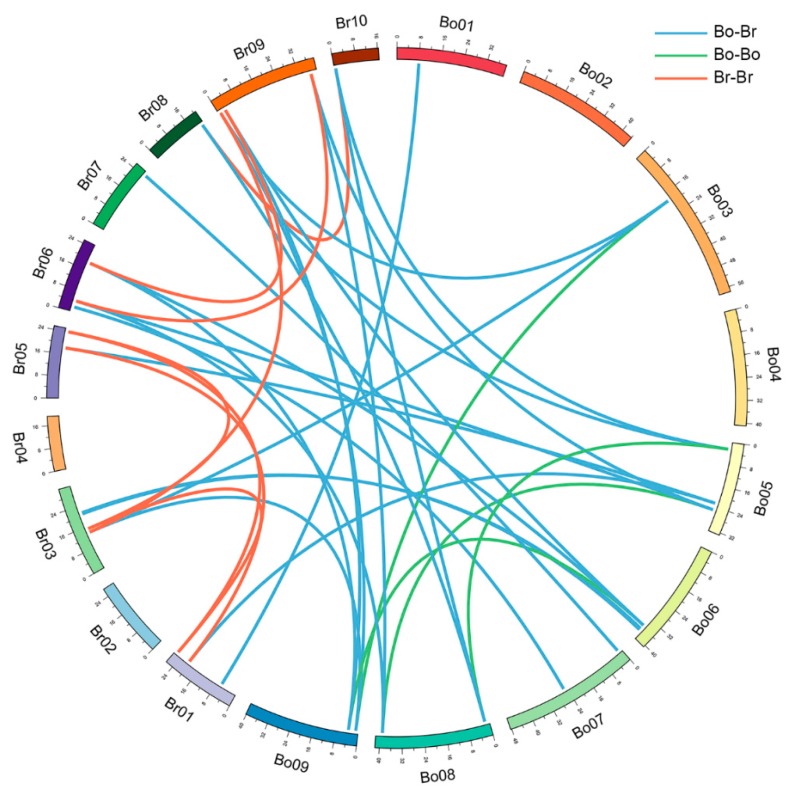
Syntenic relationship of *STP* genes shown on chromosome maps between *B. oleracea* and *B. rapa*. Nine cabbage and 10 Chinese cabbage chromosomes are shown with different random colors. Lines in blue indicate orthologous gene pairs, green indicate cabbage paralogous gene pairs, and red indicate Chinese cabbage paralogous gene pairs.

The *BoSTP* genes were distributed across the three sub-genomes (LF, the least fractionated blocks of *Brassica*; MF1, the medium fractionated blocks of *Brassica*; MF2, the most fractionated blocks of *Brassica*), with 12 *BoSTP* genes in LF, six in MF1, and four in MF2 (Table 1). Interestingly, all the *STP* genes were retained in *B. oleracea* after whole-genome triplication (WGT) and fractionation. In total, half of the *BoSTP* genes (7/14) were retained in one copy, while six *BoSTP* genes (*BoSTP1*, *BoSTP2*, *BoSTP4*, *BoSTP7*, *BoSTP9*, and *BoSTP13*) and only one (*BoSTP6*) from *B. oleracea* were retained in two and three copies, respectively (Table 1). The retained gene copies had the same conserved collinear blocks. In addition, according to the chromosomal localization of *BoSTP* genes (Figure 2), a total of 15 *BoSTP* genes (68.2%) were distributed across the nine chromosomes (C01-C09), with the exception of chromosomes C02 and C04, which had no members, and with the most (four) on chromosome C06 (Figure 2). Furthermore, seven *BoSTP* genes (*BoSTP4b*, *BoSTP5*, *BoSTP6a*, *BoSTP6b*, *BoSTP6c*, *BoSTP9b*, and *BoSTP10*) were not anchored on any of the *B. oleracea* chromosomes. The identified orthologous and paralogous *STP* genes were used to analyze the syntenic relationship between *BoSTP* and *BrSTP* genes. A total of 24 orthologous *STP* gene pairs between *B. oleracea* and *B. rapa* were identified (Figure 3). In addition, four and eight paralogous *STP* gene pairs were identified in *B. oleracea* and *B. rapa*, respectively. 

### 3.3. Ka and Ks Calculation of Orthologous STPs between Brassica oleracea and Arabidopsis thaliana

**Figure 4:**
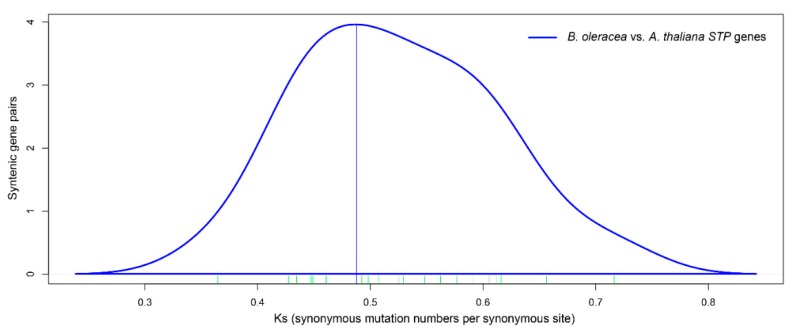
Distribution of *Ks* values of *STP* orthologous gene pairs between *B. oleracea* and *A. thaliana*.

According to the 22 syntenic STP orthologous pairs between *B. oleracea* and *A. thaliana*, the *Ks* and *Ka* values of these homologous genes were calculated by DnaSP 6 software (Table 2; Supplementary File S3). The *Ka/Ks* values of all the syntenic *STP* orthologous pairs were far less than 1, suggesting that these *STP* genes have undergone purifying selection. The *Ks* values of *B. oleracea* relative to *A. thaliana* ranged from 0.43 to 0.60 and concentrated at 0.49 (Figure 4), which indicated that these *STP* genes of *B. oleracea* diverged from *A. thaliana* approximately 16.3 MYA. We concluded that these *STP* genes diverged following the *Brassica*-specific WGT event that occurred approximately 13–17 MYA [38,56].

### 3.4. Gene Structures, TMHs, and Putative Functional Domains of BoSTP Proteins

**Figure 5:**
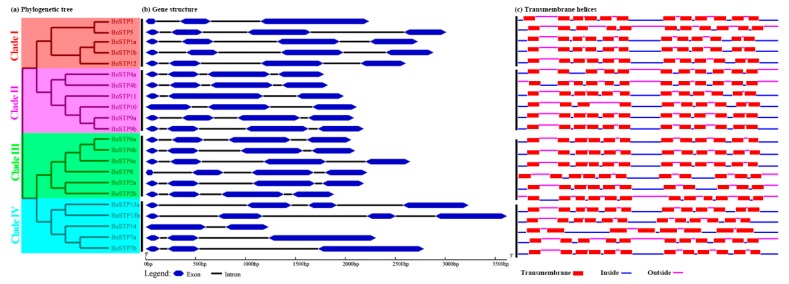
Gene structures and transmembrane helices (TMHs) of *BoSTP* genes. (**a**) An unrooted phylogenetic tree was constructed based on the full-length amino acid alignment of all the BoSTP proteins using the maximum likelihood (ML) method. (**b**) Structures of *BoSTP* genes. Exons and introns are represented by blue double-sided wedges and black lines, respectively. (**c**) TMHs of BoSTP proteins. Red rectangles signify the transmembrane regions, blue and carmine lines indicate the intracellular and extracellular regions, respectively.

The exon-intron organizations are thought to play critical roles in the evolution of multiple gene families [57]. To gain insights into the structural evolution of the *BoSTP* genes, the exon-intron boundaries were dissected. The results indicated that the number and distribution of exons and introns were highly conserved in *BoSTP* genes (Figure 5). A total of 16 (72.7%) *BoSTP* genes were found to have four exons and three introns, while five *BoSTP* members possessed three exons and two introns, and only *BoSTP14* had two exons and one intron (Figure 5). The results suggested that the gain and loss of exons might result in functional diversity of closely related *STP* genes during the evolutionary process of the *STP* gene family. Due to the significance of the sugar transporter domain for the catalytic activity, the functional domains of 22 BoSTP proteins were predicted, which revealed that all the BoSTP proteins harbored a Sugar_tr domain (PF00083) belonging to the clan major facilitator superfamily (MFS; CL0015). In addition, all the BoSTP proteins except BoSTP14 harbored an MFS domain (MFS_1 domain; PF07690) (Supplementary File S4). Among these BoSTP proteins, 15 harbored 12 conserved TMHs, five harbored 11 TMHs, and BoSTP10 and BoSTP14 harbored 10 and eight TMHs, respectively (Figure 5c). The majority of BoSTP proteins contained 12 TMHs, which is a typical feature of MFS, suggesting the activities were related to substance transportation [58].

### 3.5. Prediction of cis-Acting Elements of BoSTP Genes

In this study, the 1500-bp promoter sequences of 14 *BoSTP* genes were obtained. For the remaining eight *BoSTP* genes, the obtained promoter sequences were shorter than 1500 bp due to the presence of other genes located <1500 bp upstream. The PlantCARE web tool (http://bioinformatics.psb.ugent.be/webtools/plantcare/html/) was used to predict the *cis*-acting elements. As shown in Table 3, a total of 17 *cis*-acting elements were predicted in the promoters of the 22 *BoSTP* genes. The common *cis*-acting elements included those responsive to distinct plant hormones (ABA, auxin, ethylene, gibberellin, meJA, and salicylic acid) and stress factors (anaerobic, drought, light, low temperature, and wound) (Table 3). Among these elements, eight common *cis*-acting elements, including ABRE, ARE, CGTCA-motif, ERE, G-box, GT1-motif, STRE, and TGACG-motif, were highly conserved.

### 3.6. Expression Profiling of BoSTP Genes in Different Organs and in Response to P. brassicae Infection

**Figure 6:**
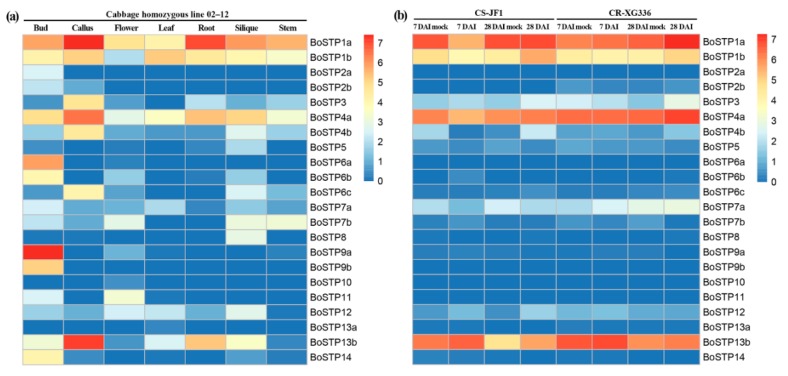
Expression profiles of *BoSTP* genes. (**a**) Gene expression patterns of *BoSTP* genes in different organs (bud, callus, flower, leaf, root, silique, and stem). (**b**) Expression dynamics of *BoSTP* genes in cabbage roots following *Plasmodiophora brassicae* infection. Expression levels of the *BoSTP* genes are shown as the log_2_ transformed FPKM (fragments per kilobase of exon model per million mapped fragments) values obtained from the RNA-Seq data. DAI: days after inoculation.

Organ-specific gene expression can provide valuable information about the function of *BoSTPs* in different organs. Thus, the transcript levels of the *BoSTP* genes in the bud, callus, flower, leaf, root, silique, and stem of cabbage derived from the RNA-Seq dataset (GSE42891) were analyzed. The heatmap revealed that a large portion of *BoSTP* genes were organ-specific (Figure 6a; Supplementary File S5). For example, several *BoSTP* genes were expressed in only one or two organ types, such as *BoSTP2a* in buds, *BoSTP13a* in leaves, *BoSTP2b* in buds and calli, and *BoSTP6a* and *BoSTP10* in buds and flowers (Figure 6a). A total of seven *BoSTP* genes were expressed in all the organs, including *BoSTP1a*, *BoSTP1b*, *BoSTP4a*, *BoSTP4b*, *BoSTP7a*, *BoSTP12*, and *BoSTP13b*. The diversity of the expression profiles of *BoSTP* genes suggested a broad range of biological functions during the growth and development of cabbage.

We also generated expression profiles of *BoSTP* genes in roots of CR-XG336 (clubroot-resistant line) and CS-JF1 (clubroot-susceptible line) infected by *P. brassica*, and observed the expression variation of *BoSTP* genes between the two different cultivars at two different infection stages (Figure 6b; Supplementary File S6). A total of five *BoSTP* genes (*BoSTP2a*, *BoSTP6a*, *BoSTP9b*, *BoSTP10*, and *BoSTP11*) were undetectable in both CR-XG336 and CS-JF1 under the two infection stages. In CS-JF1, compared with mock-inoculated plants, two *BoSTPs* (*BoSTP4b* and *BoSTP12*) and two *BoSTPs* (*BoSTP1a* and *BoSTP1b*) were significantly up- and down-regulated at 7 DAI and 28 DAI, respectively. No *BoSTP* genes were significantly up- or down-regulated in CR-XG336 at 7 DAI compared with mock-inoculated plants (Figure 6b; Supplementary File S6). Moreover, only the *BoSTP3* gene was significantly up-regulated in CR-XG336 after infection at 28 DAI (Figure 5b; Supplementary File S6).

### 3.7. Subcellular Localization Analysis of BoSTP Proteins in Tobacco

**Figure 7:**
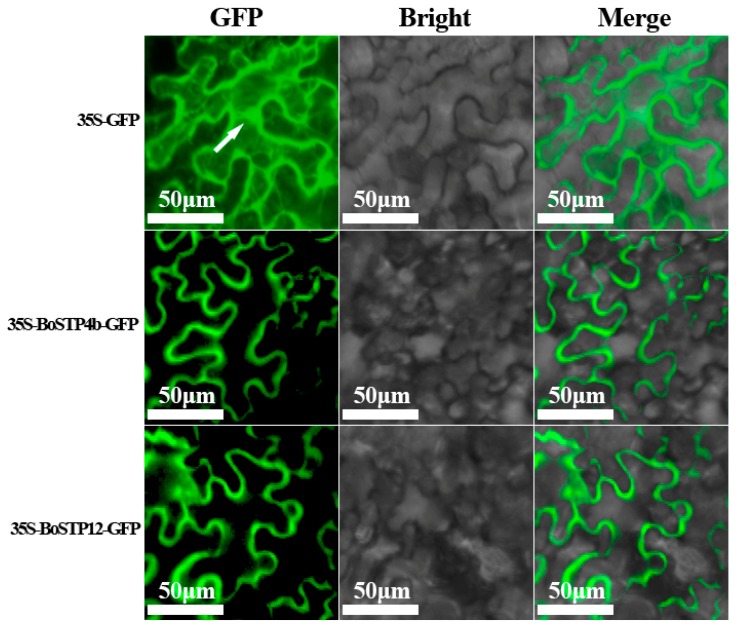
Subcellular localization of BoSTP proteins in *Nicotiana benthamiana* leaves. BoSTP4b-GFP and BoSTP12-GFP fusion proteins, as well as GFP alone, were transiently expressed in *N. benthamiana* leaves using *Agrobacterium tumefaciens* infiltration. Protein localization was examined 48 h after infiltration and representative images are shown.

In silico subcellular localization prediction using Plant-mPLoc [50] suggested that all the BoSTP proteins were localized in the cell membrane. To further characterize the subcellular localization of BoSTP proteins, BoSTP4b and BoSTP12 translational GFP fusion proteins were heterologously and transiently expressed in tobacco leaf epidermal cells. The GFP signal in epidermal cells transfected with the 35S-GFP was mainly found in the cell membrane, cytoplasm, and nucleus (Figure 7), whereas the GFP signals in epidermal cells transfected with 35S-BoSTP4b-GFP and 35S-BoSTP12-GFP fusion proteins were detected in the cell membrane (Figure 6), suggesting that the two BoSTPs are membrane proteins, consistent with the in silico prediction results.

## 4. Discussion

The availability of the *B. oleracea* genome sequence has provided great opportunity to explore the *STP* gene family members, and to investigate their phylogenetic relationships and potential functional roles. The *STP* gene family is widely distributed in the plant kingdom and plays critical roles in sugar transport, plant growth, and plant responses to pathogen attack and wounding [12,25,59]. To our knowledge, no systematic investigations on the *STP* gene family of *B. oleracea* have been reported, and the expression profiles and functional significances of the *BoSTP* genes should be further investigated.

In this study, we found that the number of *STP* genes among *B. oleracea, B. rapa*, and *A. thaliana* was variable, with 14 genes in *A. thaliana* and 22 in both *B. oleracea* and *B. rapa*. The number of *BoSTP* and *BrSTP* genes were more than that of *AtSTP* genes, possibly due to the *Brassica*-specific WGT and fractionation events [38]. Gene duplication is considered as a primary driving force for evolution, resulting in functional divergence and diversification [60,61]. In this study, we found evidence of gene replication and gene loss during polyploid speciation in the *B. oleracea* genome. After the split from *A. thaliana*, there were seven *BoSTP* genes with one separate orthologous gene, six *BoSTP* genes with two separate orthologous genes, and only one *BoSTP6* gene with three separate orthologous genes (Table 1; Figure 1). These results may indicate functional redundancy among several *BoSTP* genes.

To investigate the extent of gene fractionation in sub-genomes of *B. oleracea* relative to *A. thaliana*, three sub-genomes of *B. oleracea* were established [37]. In this investigation, a total of 12 *BoSTP* genes belonged to the LF sub-genome, six belonged to the MF1 sub-genome, and four belonged to the MF2 sub-genome (Table 1). The results indicated that the LF sub-genome contains the most *BoSTP* genes, consistent with that the LF sub-genome retains 70% of the genes found in *A. thaliana* [38]. All the BoSTP proteins harbored the Sugar_tr domain, and the majority of BoSTP proteins contained 12 TMHs, a typical feature of the major facilitator superfamily (MFS). The 12-TMH structure of STP proteins has evolved from an ancestral six-TMH transporter following gene duplication and fusion [58]. The *STP* family members belonging to MFS transporters generally possess a large central loop within the two transmembrane domains, the N-domain (TMH1–TMH6) and the C-domain (TMH7–TMH12) [11,14], and transmembrane transport is driven by the proton motive force [62]. A total of 17 *cis*-acting elements responsive to plant hormones and various stresses were predicted in the upstream sequences of the 22 *BoSTP* genes. Furthermore, the identified *cis*-acting elements, such as G-box, GT1-motif, and MRE, were transcriptionally regulated under light responsiveness, consistent with the functions of *STP* genes in sugar allocation between sink and source organs [17]. The *STP* genes play critical roles in the distribution of monosaccharides, which were found to be involved in various metabolic processes during plant growth and development [63].

For the *BoSTP* gene members, we were particularly interested in those that might play crucial roles in clubroot disease responses. Previous studies revealed that *P. brassicae* obtains sugars from hosts to complete its life cycle, involving the development and formation of galls, which act as an additional sink [64]. In *A. thaliana,* it has been found that sucrose accumulates in uninfected leaves, but not in *P. brassicae* infected leaves because sucrose is exported from leaves into the clubroot galls [65]. The expression of sugar transporter genes may therefore influence plant-*P. brassicae* interactions. In this study, the RNA-Seq dataset (PRJNA453960) of cabbage were further analyzed for dissecting the expression profiles of the *BoSTP* genes. The expression of *BoSTP4b* and *BoSTP12* were up-regulated in roots of the susceptible CS-JF1 upon *P. brassicae* inoculation at the clubroot formation stage (28 DAI) compared with mock-inoculated plants (Figure 5; Supplementary File S6). We inferred that the two *BoSTP* genes might play roles in sugar partitioning of clubroot-induced gall development and formation in CS-JF1. In *Arabidopsis*, the expression of the hexose transporter gene, *AtSTP4*, and the cell wall invertase gene, *Atβfruct1*, are induced after the powdery mildew infection. At the meantime, the uptake of glucose in host organs is enhanced substantially. The coordinated expression of *AtSTP4* and *Atβfruct1* might functionally interact for the supply of sink organs with hexoses [25]. In addition, the expression of *AtSTP8* (transporting hexose) and *AtSTP13* (transporting galactose) are up-regulated after infection. *AtSTP8* and *AtSTP13* are associated with programmed cell death in pathogen defense and cell wall remodeling [20,64]. We also noted that the expression of the *BoSTP3* gene was 3.68-fold higher in CR-XG336 at 28 DAI than in the mock-inoculated plants (Supplementary File S6). Whether this increased expression of *BoSTP3* contributes to CR-XG336 resistance to *P. brassicae* infection remains to be established.

## 5. Conclusions

In this study, a total of 22 *BoSTP* genes were identified in the *B. oleracea* genome and they were further classified into four clades based on a phylogenetic tree of 58 *STP* homologs from *B. oleracea*, *B. rapa*, and *A. thaliana*. The length of the BoSTP proteins ranged from 345 to 532 AA, and the *Ks* values of the orthologous *STP* genes in *B. oleracea* relative to *A. thaliana* ranged from 0.43 to 0.60 and concentrated at 0.49, suggesting that the estimated time of *B. oleracea* diverged from *A. thaliana* was approximately 16.3 MYA. RNA-Seq data analysis of seven organs in cabbage indicated that a large number of *BoSTP* genes exhibited organ-specific expression. The expression of two *BoSTP* genes (*BoSTP4b* and *BoSTP12*) were up-regulated in CS-JF1 at 28 DAI with *P. brassicae* infection compared with mock-inoculated plants, and we hypothesized that they might be involved in monosaccharide unloading and partitioning for the clubroot development and formation during *P. brassicae* colonization. These results help to understand the characteristics of *BoSTP* genes and their potential functions in regulating monosaccharide distribution during the clubroot disease responses in cabbage.

## Figures and Tables

**Table 1:** *STP* family genes in *Brassica oleracea* and corresponding orthologs in *Arabidopsis thaliana*.

Group	tPCKChr	CCB	*Arabidopsis thaliana*				*Brassica oleracea*											
			Gene Name	Gene ID	ORF (bp)	PL (aa)	Gene Name	Gene ID	Chromosome	Subgenome	ORF (bp)	PL (aa)	MW (kD)	pI	Instability Index	Aliphatic Index	GRAVY	SCLpred
Clade I	tPCK1	A	*AtSTP1*	*AT1G11260*	1569	522	*BoSTP1a*	*Bol036657*	C05	LF	1569	522	57.60	9.22	38.45	102.87	0.49	cell membrane
							*BoSTP1b*	*Bol031302*	C08	MF2	1566	521	57.47	9.29	39.98	102.15	0.48	cell membrane
	tPCK7	X	*AtSTP3*	*AT5G61520*	1545	514	*BoSTP3*	*Bol017006*	C06	LF	1512	503	54.76	9.12	40.17	100.95	0.45	cell membrane
	tPCK1	B	*AtSTP5*	*AT1G34580*	1521	506	*BoSTP5*	*Bol009461*	Scaffold000233	MF1	1521	506	54.43	9.28	30.06	109.13	0.69	cell membrane
	tPCK4	U	*AtSTP12*	*AT4G21480*	1509	502	*BoSTP12*	*Bol028422*	C01	LF	1542	513	56.62	8.70	30.38	106.57	0.56	cell membrane
Clade II	tPCK2	F	*AtSTP4*	*AT3G19930*	1545	514	*BoSTP4a*	*Bol018147*	C05	LF	1545	514	57.12	8.72	38.81	102.80	0.58	cell membrane
							*BoSTP4b*	*Bol000550*	Scaffold000531	MF1	1545	514	56.99	8.99	38.20	102.80	0.56	cell membrane
	tPCK1	C	AtSTP9	AT1G50310	1554	517	*BoSTP9a*	*Bol010165*	C07	LF	1551	516	56.27	6.89	32.15	99.01	0.53	cell membrane
							*BoSTP9b*	*Bol014014*	Scaffold000180	MF2	1551	516	56.15	6.89	33.77	101.67	0.54	cell membrane
	tPCK2	F	*AtSTP10*	*AT3G19940*	1545	514	*BoSTP10*	*Bol000549*	Scaffold000531	MF1	1536	511	56.16	8.60	30.51	97.50	0.48	cell membrane
	tPCK7	Q	*AtSTP11*	*AT5G23270*	1545	514	*BoSTP11*	*Bol017091*	C06	LF	1569	522	57.17	5.79	33.58	105.50	0.55	cell membrane
Clade III	tPCK1	A	*AtSTP2*	*AT1G07340*	1497	498	*BoSTP2a*	*Bol023380*	C08	MF1	1497	498	55.48	9.27	38.43	105.52	0.50	cell membrane
							*BoSTP2b*	*Bol041122*	C05	LF	1500	499	55.42	9.29	33.25	105.51	0.52	cell membrane
	tPCK2	F	*AtSTP6*	*AT3G05960*	1524	507	*BoSTP6a*	*Bol002474*	Scaffold000387	MF1	1524	507	55.86	8.76	31.67	109.98	0.65	cell membrane
							*BoSTP6b*	*Bol002866*	Scaffold000372	LF	1515	504	55.59	8.29	31.06	109.50	0.65	cell membrane
							*BoSTP6c*	*Bol034076*	Scaffold000040	MF2	1527	508	55.71	8.33	30.72	112.48	0.61	cell membrane
	tPCK7	Q	*AtSTP8*	*AT5G26250*	1524	507	*BoSTP8*	*Bol022297*	C06	LF	1452	483	53.34	8.94	32.79	112.63	0.64	cell membrane
Clade IV	tPCK5	O	*AtSTP7*	*AT4G02050*	1542	513	*BoSTP7a*	*Bol030687*	C03	MF1	1542	513	55.76	9.03	32.99	105.89	0.47	cell membrane
							*BoSTP7b*	*Bol011395*	C09	LF	1527	508	55.09	8.83	35.14	110.18	0.53	cell membrane
	tPCK7	Q	*AtSTP13*	*AT5G26340*	1581	526	*BoSTP13a*	*Bol022294*	C06	LF	1536	511	55.95	9.31	34.63	112.94	0.59	cell membrane
							*BoSTP13b*	*Bol032462*	C09	MF2	1599	532	58.04	9.30	32.58	106.65	0.49	cell membrane
	tPCK6	E	*AtSTP14*	*AT1G77210*	1515	504	*BoSTP14*	*Bol027572*	C07	LF	1038	345	38.33	8.47	45.45	112.46	0.51	cell membrane

NottPCK Chr: Chromosome of translocation Proto-Calepineae Karyotype, ancestral genome of *Brassica* species, CCB: Conserved collinear block, LF: The least fractionated blocks of *Brassica*, MF1: The medium fractionated blocks of *Brassica*, MF2: The most fractionated blocks of *Brassica*, ORF: Open reading frame, PL: Protein length, MW: Molecular weight, pI: Isoelectric point, GRAVY: Aliphatic index and grand average of hydropathicity, SCLpred: Predicted subcellular localization; STP: Sugar transporter protein.

**Table 2:** Non-synonymous (*Ka*) and synonymous substitution rate (*Ks*) of orthologous *STP* gene pairs between *B. oleracea* and *A. thaliana*.

Orthologous Gene Pairs		*Ks*	*Ka*	*Ka*/*Ks*	Duplication Date (MYA)
*AtSTP1*	*BoSTP1a*	0.4982	0.0193	0.0387	16.6
	*BoSTP1b*	0.4609	0.0189	0.0410	15.4
*AtSTP2*	*BoSTP2a*	0.6117	0.0981	0.1604	20.4
	*BoSTP2b*	0.6566	0.1081	0.1646	21.9
*AtSTP3*	*BoSTP3*	0.4924	0.0737	0.1497	16.4
*AtSTP4*	*BoSTP4a*	0.4260	0.0261	0.0613	14.2
	*BoSTP4b*	0.4345	0.0336	0.0773	14.5
*AtSTP5*	*BoSTP5*	0.6055	0.0930	0.1536	20.2
*AtSTP6*	*BoSTP6a*	0.5253	0.0452	0.0860	17.5
	*BoSTP6b*	0.5766	0.0408	0.0708	19.2
	*BoSTP6c*	0.5485	0.0560	0.1021	18.3
*AtSTP7*	*BoSTP7a*	0.5290	0.0360	0.0681	17.6
	*BoSTP7b*	0.7164	0.0476	0.0664	23.9
*AtSTP8*	*BoSTP8*	0.5900	0.0573	0.0971	19.7
*AtSTP9*	*BoSTP9a*	0.4489	0.0348	0.0775	15.0
	*BoSTP9b*	0.4279	0.0392	0.0916	14.3
*AtSTP10*	*BoSTP10*	0.3646	0.0653	0.1791	12.2
*AtSTP11*	*BoSTP11*	0.6162	0.1001	0.1624	20.5
*AtSTP12*	*BoSTP12*	0.5073	0.0500	0.0986	16.9
*AtSTP13*	*BoSTP13a*	0.4475	0.0574	0.1283	14.9
	*BoSTP13b*	0.4877	0.0203	0.0416	16.3
*AtSTP14*	*BoSTP14*	0.5623	0.0300	0.0534	18.7

**Table 3:** Known hormone-responsive and stress-responsive *cis*-acting elements in the promoter regions of *BoSTP* genes.

Elements	ABRE	ARE	CGTCA-Motif	ERE	G-Box	GT1-Motif	LTR	MBS	MRE	P-Box	STRE	TATC-Box	TC-Rich Repeats	TCA-Element	TGA-Element	TGACG-Motif	WUN-Motif	Total
	ABA	Anaerobic	MeJA	Ethylene	Light	Light	Low Temperature	Drought	Light	Gibberellin	Stress Response	Gibberellin	Defense & Stress	Salicylic Acid	Auxin	MeJA	Wound	
*BoSTP1a*	2	3		2	2		1				1						1	12
*BoSTP1b*	2	4		1	3						1		1				1	13
*BoSTP2a*		2																2
*BoSTP2b*				2				1		2								5
*BoSTP3*	1	2			1													4
*BoSTP4a*					1		1			1								3
*BoSTP4b*			3	1				1							2			7
*BoSTP5*	1	1	1		1	1								2		1	1	9
*BoSTP6a*	1				1													2
*BoSTP6b*	1	1			2	3	3										1	11
*BoSTP6c*		2		3		1			2		1		1		3			13
*BoSTP7a*	2		2	4	3	1					1	1		1		2	1	18
*BoSTP7b*	4		2	1	6						1	1		1		2	1	19
*BoSTP8*		3	2	1				1		3						2	1	13
*BoSTP9a*	2	3	1	4	2	1					2		2		2	1		20
*BoSTP9b*		5	1			3	2			1	1		2			1		16
*BoSTP10*	2			3	2	2					4						1	14
*BoSTP11*	1		1	2	1			2	1		1			1		1		11
*BoSTP12*	2	1			2	2			1					1				9
*BoSTP13a*	5	2	1	2	6			1		1	1	1			1	1		22
*BoSTP13b*	3	2	2	1	4	2		1				1				2	1	19
*BoSTP14*		3	1			1					1	3		1		1		11

## Supplementary Materials

The following are available online at http://www.mdpi.com/2073-4425/10/1/71/s1, Supplementary File S1: Primers used in this study; Supplementary File S2: Amino acid sequences of 58 STP proteins from *B. oleracea*, *B. rapa* and *A. thaliana*; Supplementary File S3: Nucleotide sequences of 58 *STP* genes without stop codons of *B. oleracea*, *B. rapa* and *A. thaliana*; Supplementary File S4: Predicted functional domains of BoSTP proteins; Supplementary File S5: Expression of *BoSTP* genes in different organs including bud, callus, flower, leaf, root, silique and stem in homozygous cabbage line 02-12; Supplementary File S6: Expression of *BoSTP* genes in roots of clubroot-resistant and susceptible cabbage lines at two different *P. brassicae* infection stages.
